# Asymmetrical fatiguing of the gluteus maximus muscles in the elite short-track female skaters

**DOI:** 10.1186/s13102-020-00193-w

**Published:** 2020-08-14

**Authors:** Mariusz Konieczny, Paweł Pakosz, Mateusz Witkowski

**Affiliations:** 1 Faculty of Physical Education and Physiotherapy, Opole University of Technology, Prószkowska 76, 45-758 Opole, Poland; 2 School of Physical Education and Sport, Adam Mickiewicz University in Poznań, Królowej Jadwigi 27, /39 61-871 Poznań, Poland

**Keywords:** Electromyography, Motor laterality, Short-track, Muscle myolectrical manifestations of fatigue

## Abstract

**Background:**

According to research, fast skating on short distances increases functional asymmetry of leg muscles. As has been proven in many sporting disciplines, this asymmetry can increase the risk of injury. The aim of the study was to analyze the level of right and left myolectrical manifestations of fatigue asymmetry of gluteus maximus muscle in elite skaters on a short track and to compare this phenomenon to a control group. The muscles were chosen deliberately, due to their influence in maintaining the right position during training on ice.

**Methods:**

The experiment compared a group of eight members of the Polish Women’s National Team in short track with a group of eight non-training people. The subjects did the Biering-Sorensen test, in which sEMG (surface electromyography) signal frequency was measured in the gluteus maximus muscles during an isometric contraction. Myolectrical manifestations of fatigue slopes were analyzed with a ANOVA with repeated measures. In the skaters, the myolectrical manifestations of fatigue differed between the right and the left gluteus maximus muscles. All the skaters had higher myolectrical manifestations of fatigue in the right leg. This phenomenon was not observed in the non-training subjects, who on average had similar myolectrical manifestations of fatigue in both legs.

**Results:**

Results showed that the right and the left muscles of the skaters in the experimental group differed in myolectrical manifestations of fatigue, but this difference was non-significant in the control group.

The subjects from the two groups did not differ in the myolectrical manifestations of fatigue of the left muscle, they did in the myolectrical manifestations of fatigue of the right muscle. The elite speed-track skaters had higher myolectrical manifestations of fatigue in the right muscle than the non-training subjects.

**Conclusions:**

Training should thus be planned in a way that minimizes the risk of causing muscle myolectrical manifestations of fatigue asymmetry in skaters despite the typically asymmetrical muscle work during training on ice and competition, thus new training protocols should be developed or considered to decrease that asymmetry.

**Trial registration:**

The tests were previously approved by the Bioethical Commission of the Chamber of Physicians in Opole. (Resolution No. 235 of 13 December 2016).

## Background

Muscular asymmetry, especially in professional athletes, increases the risk of injury, as pointed out by various authors examining asymmetry of muscles in football players, basketball players, and people with spinal pains [1–4]. Surface electromyography is considered a reliable and credible tool for assessing the post-effort myolectrical manifestations of fatigue of muscles. In this kind of analysis, the most often used parameters of the sEMG signal are changes in amplitude scope and in the mean or median frequency of total capacity spectrum. In some studies however physical effort did not decrease the median frequency sEMG signal [5–8].

Many authors have used the Biering-Sorensen test to examine a myolectrical manifestations of fatigue level and determine differences in the muscular work of symmetrical muscles [9–11]. To meet the needs of analysing muscular myolectrical manifestations of fatigue in different body positions, various elements of the Biering-Sorensen test have been modified, such as body position and the time of conducting the test [12, 13]..

The asymmetry of gluteus maximus muscles has been described in the literature most often in the contexts of walk and isolated positions [11, 14]. In short track, previous research has dealt with the muscular work of only one limb or with asymmetry in the myolectrical manifestations of fatigue of muscles during skating. Felser et al. studied athletes skating in a straight line and in curves [15]. Neuromuscular activation was higher in the right leg, while with the reduction in skating speed decreased neuromuscular activity, but only when skating in a straight line. This indicates that the right leg has higher activity during skating in curves. Studying athletes skating in a straight line and in curves during the subsequent laps, Hesford et al. found considerable asymmetry in oxygen supply to the two legs. The authors did not report effects of this asymmetry, but offered suggestions for training [16]. Stoter et al. showed that the bio-electric tension of muscles of speed skaters was correlated with speed at different sections of the track, but they did not analyse whether muscular asymmetry affected this phenomenon [17]. At present, the literature of the examined phenomenon contains analyses of muscle myolectrical manifestations of fatigue in many variants [18]. Non-invasive methods of determination of myolectrical manifestations of fatigue parameters also include surface electromyography (sEMG). Analysis of the sEMG signal frequency of the power spectrum provides useful information concerning local muscle myolectrical manifestations of fatigue [7, 19].

The idea behind the study was initiated by the coaches of the Polish National Team, worried about the asymmetry in the skaters and the related increased injury risk. Even though the coaches try to focus as much of the training as possible on symmetrical work, they stress that training on ice accounts for about 60% of the training volume and skating to the left is a typical asymmetrical work. Since the level of asymmetry varies from athlete to athlete, the coaches stress the importance of customised training, which would help them improve muscular symmetry in each athlete in an optimal way.

The main research hypothesis is that intensive short-track training leads to asymmetry of the gluteus maximus muscles. Thus, the paper aims to study the size of asymmetry in muscle activity and assess the difference in fatigability between the right and the left gluteus maximus muscles of the Polish Women’s National Team in short track, and to compare this fatigability with that in non-training women.

## Methods

### Participants

Two research groups took part in the tests. The experimental group included eight female members of the Polish National Team in short track, with a mean age of 18.7 ± 2.9 standard deviation, mean height of 162.4 ± 2.4 cm, and mean body weight of 57.2 ± 5.9 kg. The control group included eight female students active in sports (but not in speed skating), with a mean age of 20 ± 0.9, mean height of 169.1 ± 4.1 cm, and mean body weight of 68 ± 4.2 kg. These students were randomly selected from among female students of physical education at Opole University of Technology. The research was conducted during the training cycle, after a weekend break in training, to avoid the short-term effect of myolectrical manifestations of fatigue accumulation due to the training. The participants were informed about the purpose and course of tests and signed a consent to participate in the tests. The tests were approved by the Bioethical Commission of the Chamber of Physicians in Opole, Poland. In interviews conducted before the tests, all the respondents declared they were right-handed and right-legged in daily and sports activities (e. g., tossing a ball, kicking a ball, supporting with a foot during swinging). Furthermore, a kick-a-ball test (with three attempts) confirmed all the participants were right-legged, while the modified Edinburgh questionnaire confirmed they were right-handed [20].

### Procedures

**Fig. 1:**
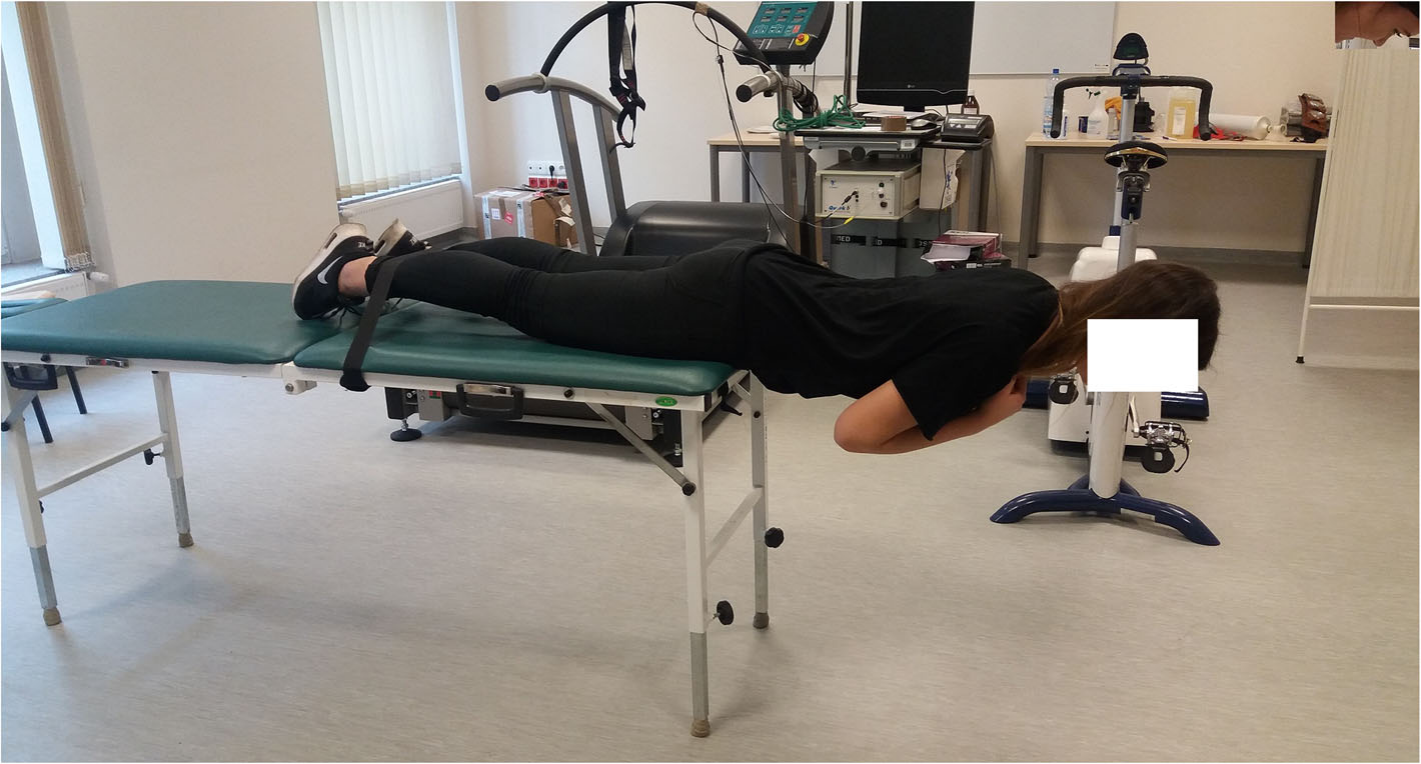
Body position in the Biering-Sorensen test

The sEMG signal frequency in the gluteus maximus muscles was examined in an isometric contraction using the position from the Biering-Sorensen test [11, 12]. To avoid too high loads for the skaters, the tests were stopped after 60 s of the contraction, and they were not continued until the subject was unable to hold the position because of muscle myolectrical manifestations of fatigue. The effectiveness of the myolectrical manifestations of fatigue test during a 60-s contraction was confirmed by Mutchler et al. [21]. During the test, the subjects were lying on a horizontal table on the abdomen, with the iliac crests aligned to the edge of the table and the lower limbs attached to the straps around the ankle joints. They were instructed to hold the body (head, shoulders, and torso), without support, horizontally to the ground as long as they could, with the arms crossed at the chest (Fig. 1).

### The EMG measurement

In the test, a 16-channel EMG system (produced by NORAXON DTS) was used, which recorded signals with an accuracy of 16 bits at a sampling rate of 1500 Hz. The bio-electric test of activity of the right and the left gluteus maximus muscles was carried out by the SENIAM methodology [11, 22]. To improve the adherence of the electrodes, before the test, the hair was shaved and the skin was cleaned in the place where the electrodes were to be stuck. Surface electrodes (Ag/AgCl) were placed on the muscle between the movement point and the tendon attachment, along the longitudinal middle line of the muscle. Signal processing and EMG analysis were performed using NORAXON MR-XP 1.07 Master Editionx software.

**Fig. 2:**
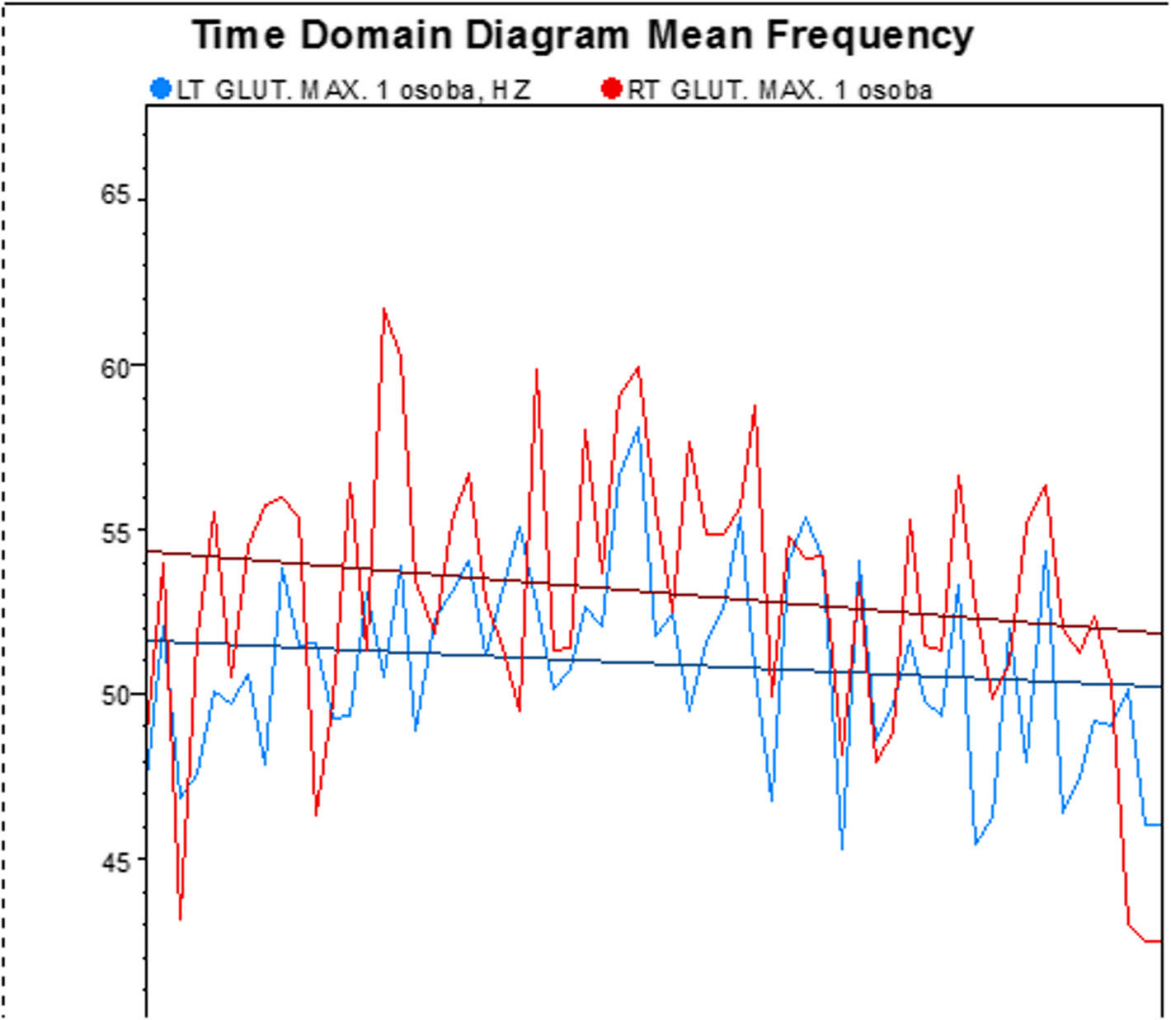
Exemplary raw sEMG records during warm-up before testing the Biering-Sorensen test

**Fig. 3:**
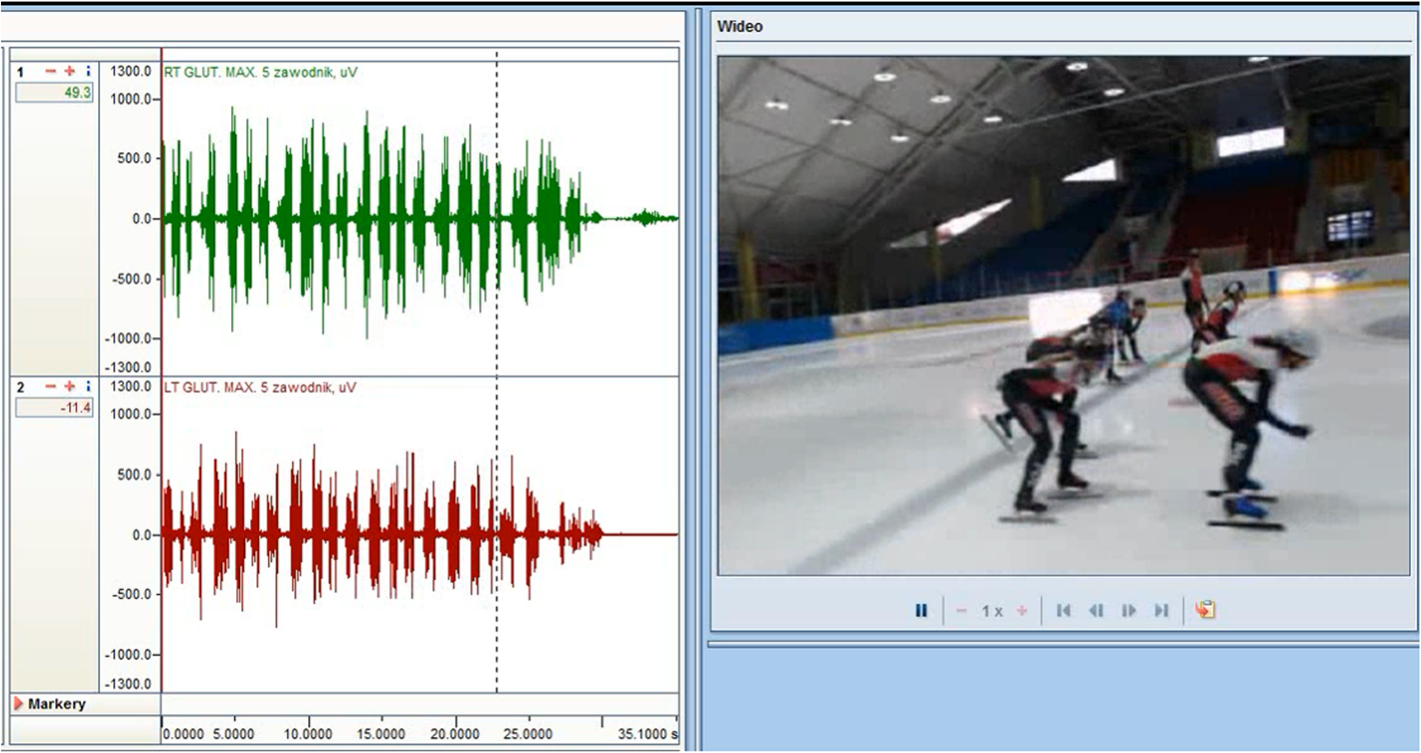
Exemplary myolectrical manifestations of fatigue slope (raw sEMG records) being a regression coefficient from a linear regression line between the mean frequency and time

Myolectrical manifestations of fatigue-related changes (frequency shift) in the frequency content were calculated for the raw EMG signal (Fig. 2) obtained during a static contraction. Unfiltered raw sEMG was analysed step-wise in 1000 ms increments over the selected portion of the measurement (60 s in the Biering-Sorensen test). The mean frequency was calculated for each step using values based on the frequency power spectrum (calculated by a Prime Factor Fourier Transformation). A myolectrical manifestations of fatigue slope (being a regression coefficient from a linear regression line between the mean frequency and time) was estimated for each participant. The mean value of the slopes of these lines were analyzed with mixed ANOVA within-between interactions taking into account the side factor (left/right muscle) and the studied group (experimental, control). sEMG frequency power spectrum is expected to shift to lower frequencies during fatiguing contractions, and the mean frequency analysis can be used to estimate the magnitude of that shift. This phenomenon is well established for static contractions at constant load levels and believed to reflect local myolectrical manifestations of fatigue (Fig. 3.).

Technical specification of NORAXON DTS is as follows:

- basic noise of the device, below 1 uV RMS,
- input impedance above 100 Momh,
- CMR (common signal rejection factor) greater than 100 dB,
- sampling frequency 1500 Hz,
- gain500.

### Statistical analysis

The slopes representing the subjects’ myolectrical manifestations of fatigue were analyzed with mixed ANOVA within-between interactions. The two groups (short track and control) constituted the between-subject factor, and the two sides (left leg and right leg) constituted the within-subject factor. Since the interaction was significant, Tukey’s post hoc tests were applied for pair-wise comparisons of the four factor combinations. For the analyses, a 0.05 significance level was used. All analyses were made in Statistica v. 13.1.

## Results

**Fig. 4:**
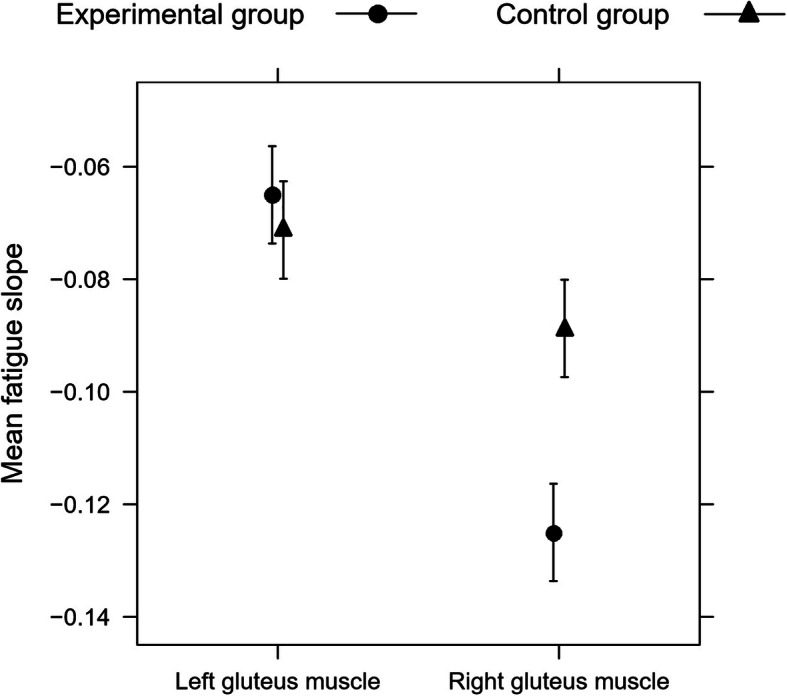
Mean myolectrical manifestations of fatigue slopes for the right and the left gluteus maximus muscles of the skaters and non-training people (means ± standard error of the mean from the linear model)

In the ANOVA, the main effect of the group was non-significant (*F* (1, 14) = 2. 964, *p* = 0.107). The main effect of the side (right-left) of the muscle, however, was significant (*F* (1, 14) = 20.323, *p* < 0.001), and so was the group-by-side interaction (*F* = (1, 14) = 6.111, *p* = 0.0268) (Fig. 4). Tukey’s tests (Table 1) showed that the right and the left muscles of the skaters (i.e., the subjects in the experimental group) differed in myolectrical manifestations of fatigue (*p* = 0.001); this difference was non-significant in the control group. The subjects from the two groups did not differ in the myolectrical manifestations of fatigue of the left muscle, they did in the myolectrical manifestations of fatigue of the right muscle. The elite speed-track skaters had higher myolectrical manifestations of fatigue in the right muscle than the non-training subjects. Size differences are shown in Fig. 4.

**Table 1:** Post-hoc Tukey’s tests for the combinations of the groups (skaters and control) and side (left and right muscle)

	Skaters, right muscle	Skaters, left muscle	Control, right muscle
Skaters, left muscle	0.001*		
Control, right muscle	0.029*	0.234	
Control, left muscle	0.001*	0.955	0.497

*p ≤ 0,05

**Fig. 5:**
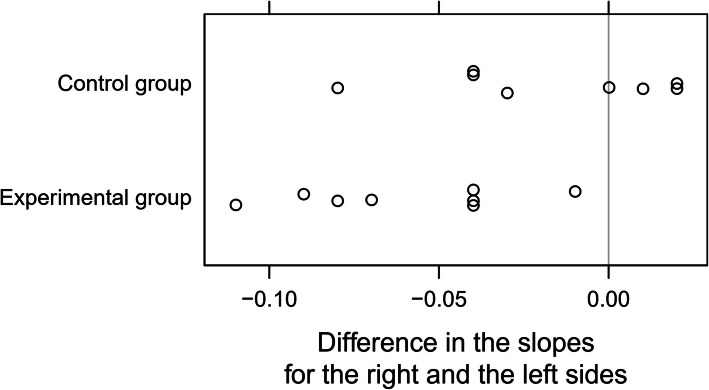
The differences in the myolectrical manifestations of fatigue slopes between the right and the left gluteus maximus muscles for each subject from the two groups studied. Slight vertical jitter (random noise) was added to avoid the overlap of the points. The grey vertical line represents a no-difference reference point

Figure 5 shows significant interactions that indicate that the average slope differences between the right and left muscles differ between the two groups. The more to the left a point lies, the greater the difference between the subject’s muscles was. All elite skaters, the asymmetry is visible in the graph (so the points lie far to the left from the zero line). In a non-trained group only a several people had this asymmetry (so only a few points lie far to the left of the zero line). We see thus that the interaction was significant because the groups differed in these differences (representing asymmetry) in the myolectrical manifestations of fatigue of the right and the left muscles.

**Fig. 6:**
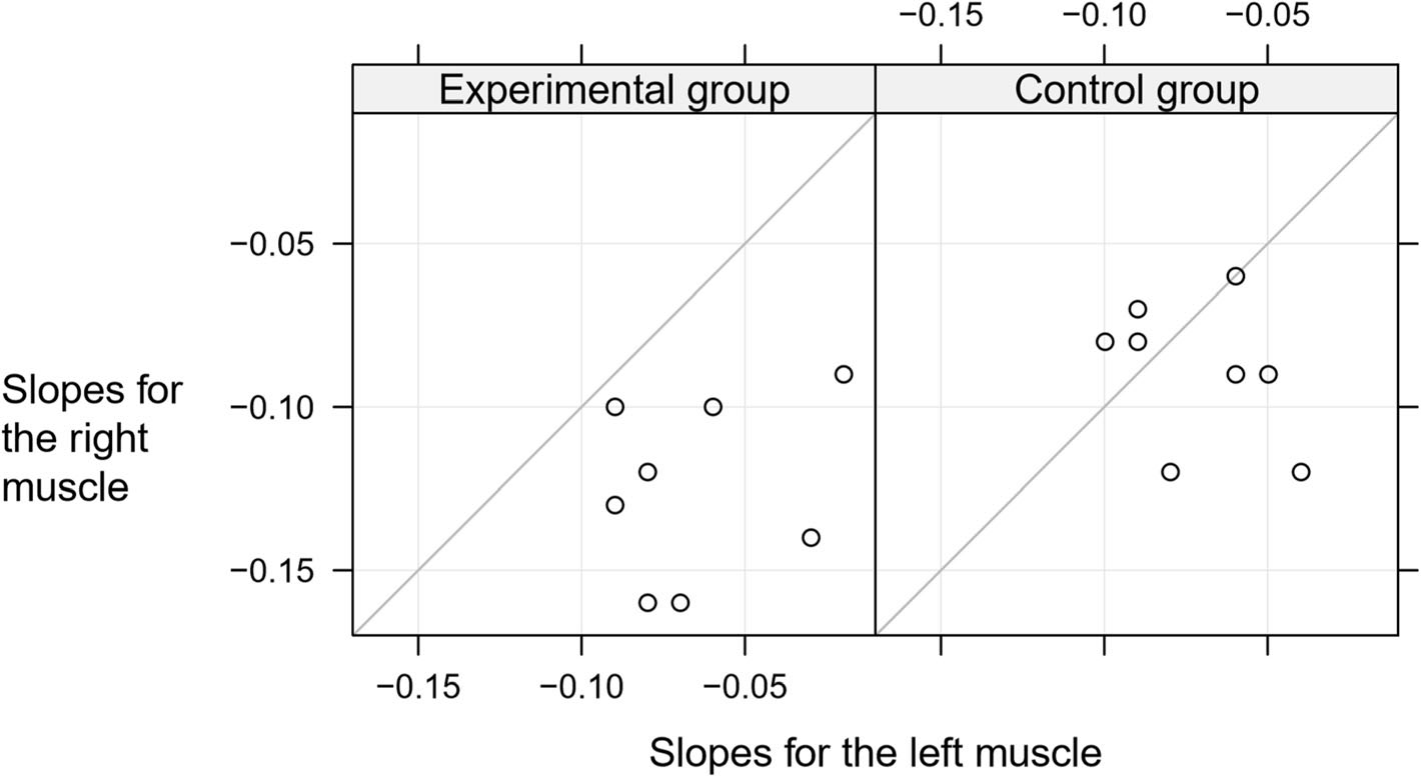
The myolectrical manifestations of fatigue slopes estimated for the right gluteus maximus muscle versus the slopes estimated for the left muscle, for the two groups. The grey diagonal reference lines represent no difference between the myolectrical manifestations of fatigue slopes for the two muscles. To facilitate the muscle-to-muscle comparison, the vertical and horizontal scales share the minimum and maximum values and have the isometric property. A subject with small myolectrical manifestations of fatigue in both muscles would be represented by a point located in the top right corner of the corresponding panel; a subject with severe myolectrical manifestations of fatigue in both muscles would be represented by a point located in the bottom left corn

Figure 6 pairs the left and right maximus gluteus muscles for each subject. This graph clarifies that the non-training subjects who had a small difference in the myolectrical manifestations of fatigue of the muscles had in general low myolectrical manifestations of fatigue of both muscles. While the subjects from the two groups did not differ in the myolectrical manifestations of fatigue of the left muscle, they did in the myolectrical manifestations of fatigue of the right muscle: the elite speed-track skaters had higher myolectrical manifestations of fatigue in the right muscle, and higher than the non-training subjects (*p* = 0.001; Table 1 and Figs. 4 and 5).

## Discussion

The research aimed to determine differences in muscle myolectrical manifestations of fatigue in the left and the right maximum gluteus muscles in female short-track skaters and compare these differences with those in healthy non-training women.

What was surprising, however, was so high asymmetry of muscle myolectrical manifestations of fatigue observed in the members of the Polish Women’s National Team in short-track speed skating. In general, short-track training focuses on symmetrical movements, so it should not generate so high asymmetry between the right and the left gluteus maximus muscles. Too high asymmetry in these muscles can increase the risk of injury, so is unfavourable in high-performance athletes [1–3]. thus its importance should not be underestimated in professional sports that involve alternating limb movements.

Such high asymmetry, as in the tested athletes, however, has not been observed in the non-training subjects. Asymmetry in an experimental group can be caused by specific training in short-track. Small asymmetry in non-training people is likely a normal phenomenon, and it should not increase the possibility of injury.

Even though experiments without the control group are difficult to interpret, most studies on muscle myolectrical manifestations of fatigue did not include non-training subjects [15, 16]. The control group was included in the experiment to analyze muscle asymmetry in skaters against the background of non-training people, which did help us draw richer conclusions about the studied phenomenon.

The studied skaters train at a skating rink in about 60% of the annual training volume, remaining 40% being focused on symmetrical training of both limbs. Various elements of their individual skating techniques—especially the technique of skating on curves—can cause asymmetry in the gluteus maximus muscles, an unfavourable phenomenon in speed skating because of the increased injury risk it poses. Unfortunately, the phenomenon of muscle asymmetry in professional short-track skaters is still poorly studied, which makes it difficult to design a training process that would not generate asymmetry. Asymmetry in muscle fatigability is certainly not limited to short track, however. Mastalerz et al. [23] reported differences in myolectrical manifestations of fatigue between the muscles of the right and the left legs in runners, reaching from 12,5% to 26,5%, depending on the muscle Hartz et al. measured the myolectrical manifestations of fatigue index through the induction of shoulder evation movement [24].

Felser et al. showed that muscle tension activity in muscles of both legs differed between the moments of skating in a straight line and in curves; greater differences occurred in the right leg muscles [15]. This result stresses the importance of the cornering technique, likely that element of the training which most strongly increases asymmetry of leg muscles. Hesford et al. also observed asymmetry in legs of though they studied the oxygenation of the quadriceps muscle [16].

### Study’s limitations

The limitation of the studies was the size of the experimental group, but due to the specificity of the group, it was not possible to study more athletes.

During the experiment, the skaters were in the middle of the training cycle. On the other hand, the tests were conducted after a weekend break in training, so the muscles were relaxed; had the tests been conducted right after training, the muscles would have been tired, which might have affected the tests. During the gluteal muscle myolectrical manifestations of fatigue research, there was no reference to the activation of other muscles to develop myolectrical manifestations of fatigue. In future studies, it is therefore worth increasing the number of muscles to better investigate the myolectrical manifestations of fatigue process. The sEMG signal frequency change analysis method we used is not a direct method to determine the myolectrical manifestations of fatigue level but only an indirect method.

## Conclusions

Elite female short-track skaters have significant asymmetry in fatigability of the gluteus maximus muscles.

In healthy individuals there is no statistically significant difference in the asymmetry of myolectrical manifestations of fatigue the gluteus maximus muscles.

The results offer an important lesson for the coaches of the Polish Women’s National Team in short-track skating.

## Data Availability

The datasets generated and/or analysed during the current study are not publicly available. However, the data are available from the corresponding author on reasonable request.

## Abbreviations

sEMG: Surface electromyography
Ag:/AgCl: Silver chloride electrode
RMS: Root-mean-square
CMR: Common signal rejection
